# Competitive Stereocomplexation and Homocrystallization Behaviors in the Poly(lactide) Blends of PLLA and PDLA-PEG-PDLA with Controlled Block Length

**DOI:** 10.3390/polym9030107

**Published:** 2017-03-15

**Authors:** Zhanxin Jing, Xuetao Shi, Guangcheng Zhang

**Affiliations:** Ministry of Education Key Lab of Applied Physics and Chemistry in Space, College of Science, Northwestern Polytechnical University, Xi’an 710072, China; jingzhan_xin@126.com

**Keywords:** PLLA, PDLA-PEG-PDLA, stereocomplexation, homocrystallization

## Abstract

Stereocomplex poly(lactide) (PLA) was obtained by solution blending of linear PLLA and PDLA-PEG-PDLA. Effects of the L/D ratios, PEG block, and PDLA block on stereocomplexation of the blends are systemically discussed. The full stereocomplex PLA can be acquired by solution blending when L/D ratios are in the range of 7/3–5/5. The experiment results demonstrated that the stereocomplex degree of PLLA/PDLA-PEG-PDLA prepared by melt blending was closely related to the PEG block and PDLA block. POM results indicated that the blends with high L/D ratio showed large disordered spherulites, and the typical Maltese cross pattern was observed as the L/D ratios decreased. The results of PEG block on the stereocomplexation of PLLA/PDLA-PEG-PDLA revealed that the PEG blocks possessed two sides: accelerating agent for the mobility of polymer chains and decreasing nucleation capacity due to their diluting effect. The effect of PDLA block on the stereocomplexation of the blends was also well investigated. The results showed that the crystallization of sc-crystallites and hc-crystallites in the PLLA/PDLA-PEG_4k_-PDLA blends with different PDLA blocks presents an obvious competition relationship, and this is not beneficial to the formation of sc-crystallites with increasing PDLA block. The melting behavior of PLLA/PDLA-PEG_4k_-PDLA with different PDLA blocks after isothermal crystallization showed that the blends could achieve full stereocomplex when the crystallization temperature exceeded 160 °C, and a crystallite with high perfection could be formed as the crystallization temperature increased. This study systemically investigated the effects of the L/D ratios, PEG block, PDLA block, and crystallization conditions on stereocomplex crystallization of PLLA/PDLA-PEG-PDLA blends, which can provide potential approaches to control the microstructure and physical performances of PLLA/PDLA-PEG-PDLA blends.

## 1. Introduction

Poly(lactide) (PLA) as a biodegradable thermoplastics polyester [1,2] has attracted the widespread attention of many researchers and is considered to be a sustainable alternative to petrochemical-derived products [3]. PLA has been widely used in many fields, such as drug delivery, tissue engineering, packaging, and fiber, due to its non-toxicity, biodegradability, and bio-compatibility [4,5,6]. However, its low crystallization rate, fast degradation rate, poor heat resistance, and toughness of PLA have limited its application in some fields. Nowadays, many methods have been used to improve the properties of PLA, such as nanocomposite technology, blending technology, cross-linking technology, and stereocomplexation [7,8,9,10]. Stereocomplexation is considered an effective method to improve the heat resistance and crystallization capacity of PLA, which is an essential issue in widening the scope of industrial and commodity applications.

A stereocomplex consists of two components containing the same composition and different three-dimensional configurations [11]. It is well known that there exists a quaternary carbon atom in lactic acid, which is the synthesis monomer of poly(lactide). Therefore, poly(lactide) could be divided into poly(l-lactide), poly(d-lactide) (PDLA), meso poly(lactide) (meso-PLA) and racemic polylactic acid (PDLLA). PLA stereocomplex (sc-PLA) could be formed by the strong van der Waals forces of CH_3_^…^C=O interactions between PLLA and its enantiomer PDLA [12]. Previous literature references [12,13] reported that the melting temperature of PLA stereocomplex could reach 230 °C, which is higher than that of homopolymer poly(lactide) (PLLA or PDLA) (about 180 °C). Sc-PLA presents a crystalline β-form, which is different from the α-form of PLLA or PDLA [14]. Furthermore, sc-PLA presents a fast crystallization rate, as well as excellent mechanical properties, degradation characteristics and heat distortion temperature compared to those of PLLA or PDLA [15]. However, Pan et al. [16] and Tsuji et al. [17] demonstrated that stereocomplexation and homocrystallization are a competitive relationship in PLLA/PDLA blends or PLLA-*b*-PDLA copolymers, so it is difficult to have the formation of a full stereocomplex due to limitation of memory to re-form stereocomplexation of PLA in the melt processing, especially for high-molecular weight PLLA or PDLA. Furthermore, the effect of molecular weight, thermal treatment, annealing, and blend composition on stereocomplex and homocrystal crystallization is the main theme of the formation of PLA stereocomplex [18,19]. Sarasua [20] reported that exclusive stereocomplexation with a high degree of crystallization from high molecular weight polylactides can be achieved by a well controlled crystallization temperature. Furthermore, the competition between PLA stereocomplexation and homocrystal crystallization is strongly related to PLA molecular weight, thermal treatment, and blend composition [18,19]. Sarasua et al. [20] reported that exclusive stereocomplexation with a high degree of crystallization from high molecular weight polylactides can be achieved by good controll of the crystallization temperature. Therefore, it is quite important to find effective technology for the stable formation of sc-PLA while maintaining its superior properties. Many methods, such as nanocomposite technology [21,22], physical blending with flexible polymer [22], as well as copolymer with a soft segment [23,24], have been used to improve the stereocomplex degree. At present, the incorporation of soft segments by copolymerization into the PLLA or PDLA chain has been known to be the most effective way to obtain the full stereocomplex PLA [23,25]. The presence of soft segments could obviously improve the mobility capacity of the PLA chain, which could facilitate the formation of sc-crystallites. Some literature references [25,26,27] have reported that soft segments, such as PEG and PCL, could accelerate the formation of sc-crystallites.

In the literature, PEG either as plasticizer or block-segment has been reported by many researchers [28,29] to improve the performance of PLLA for biomedical applications, since PEG is a nontoxic, water-soluble polymer which exhibits rapid clearance from the body, and has been applied in a vast range of biomedical applications [30]. In addition, diblock or triblock PLA-PEG copolymers were also synthesized to improve hydrophilicity and drug-delivery properties of PLA. In 2001, the PLA stereocomplexation mechanism was first introduced to prepare specifically the “thermosensitive hydrogels” from triblock copolymers PLA-PEG-PLA by Fujiwara [31]. Some studies [24,27,31,32,33] on PEG-PLLA block copolymer as compatibilizer of PLLA have been reported, revealing that the blend presents excellent properties due to softening of the PEG block. Therefore, it is worth combining the flexible PEG and stereocomplex technology to manipulate the crystallization behavior of PLA. In this study, PDLA-PEG-PDLA block copolymers with different PDLA or PEG blocks were synthesized by ring-opening polymerization, using PEG and Sn(Oct)_2_ as initiator and catalyst, respectively. A series of PLLA/PDLA-PEG-PDLA blends was then prepared by solution blending for the PLA stereocomplexation. The chemical structure, thermal properties, phase behaviors, and crystal morphology of PLLA/PDLA-PEG-PDLA blends with similar composition and different L/D ratios were investigated. Non-isothermal or isothermal crystallization behaviors of the blends with different PEG or PDLA blocks were also systemically analyzed.

## 2. Experimental Section

### 2.1. Materials and Reagents

Poly(l-lactide)(PLLA, 4032D) was purchased from Nature-works LLC (Minnetonka, MN, USA); d-lactide (optical purity >99%) were supplied by Changchun Sheng Boma Ltd. (Changchun, China); Stannous octoate (Sn(Oct)_2_, chemically pure) was bought from Sinopharm Chemical Reagent Co., Ltd. (Shanghai, China); Polyethylene glycol (PEG) with different molecular weights was purchased from J&K Chemicals (Beijing, China); Other reagents were analytical grade without further purification.

### 2.2. Synthesis and Characterization of PDLA-PEG-PDLA

Certain amounts of dried PEG and d-lactide were first added to the polymerization tube. Then the catalyst Sn(Oct)_2_ in toluene solution (10 mg/mL) was added to the tube (the weight ratio between catalyst Sn(Oct)_2_ and monomer d-lactide was 0.08:1). The tube was purged with nitrogen to eliminate air, and pumped to 30 Pa to remove residual toluene. Finally, the tube was sealed by melt-sealing the tube, and placed in an oil bath at 140 °C. After 72 h, the reaction was terminated by cooling the tube to room temperature. The obtained substance was dissolved in CHCl_3_, and precipitated using an excess of CH_3_OH. The precipitate was collected by filtration under reduced pressure. The purification process was repeated three times to remove completely Sn(Oct)_2_ and the unreacted d-lactide. Finally, the precipitate was dried in a vacuum oven for 48 h at 50 °C. The obtained substances were marked as PDLA-PEGX-PDLA Y, where X shows the molecular weight-number of PEG and Y represents the theoretical mole ratio of d-lactide to PEG in PDLA-PEG-PDLA. The special reaction conditions are listed in Table 1. The synthetic PDLA-PEG-PDLA was characterized by NMR (Bruker AV 300 NMR, Bruker BioSpin, Rheinstetten, Germany), GPC (Waters 1515, Milford, MA, USA) and DSC (Mettler DSC1, Greifensee, Switzerland), and the obtained data are listed in Table 1. The NMR spectra are shown in Figure S1.

### 2.3. Preparation of PLLA/PDLA-PEG-PDLA Blends

PLLA and PDLA-PEG-PDLA were first dissolved in dichloromethane solution, separately and both solutions were prepared with a concentration of 10 mg/mL. Then the two solutions were fully mixed by magnetic stirring for the formation of PLA stereocomplex via a solution method. The obtained solution was poured into a glass dish for two days at room temperature for the evaporation of dichloromethane. Afterwards the obtained blends were dried in a vacuum oven for 24 h at 50 °C to eliminate the residual solvent. The prepared blends were designated as PLLA/PDLA-PEG_X_-PDLA_Y_ M/N, where M and N represents the L/D mole ratio in the blends, respectively. The mixing L/D ratio of the blends was fixed at 5/5 if not mentioned otherwise.

### 2.4. Measurement of Blends

*FT-IR*. FT-IR spectra of the blends were measured by a Fourier transform infrared spectrometer (Nicolet 5700, Madison, WI, USA) in the transmission mode. The spectra were registered with 32 scans and a resolution of 4 cm^−1^.

*DSC*. (Differential scanning calorimetry) The crystallization and melting behavior of PLLA/PDLA-PEG-PDLA blends were performed on a METTLER DSC (Greifensee, Switzerland). The weighted samples (about 5–10 mg) were sealed in an aluminum pan. For PLLA/PDLA-PEG-PDLA blends with different L/D ratios, DSC curves were measured using the followed conditions: the sample was first heated from 0 to 240 °C at a rate of 10 °C/min, and kept at this temperature for 4 min to erase heat history; then it was cooled to 0 °C and reheated to 240 °C at 10 °C/min. For non-isothermal crystallization of the blends with different PEG or PDLA blocks, samples were cooled quickly to 0 °C (−50 °C/min) after melting at 240 °C for 4 min, and then heated to 240 °C at 10 °C/min. During the isothermal crystallization, samples were cooled to the desired temperature at the rate of 50 °C/min after melting at 240 °C for 4 min, and held at this temperature for sufficient time to completely crystallize. Eventually, samples were reheated to 240 °C at 10 °C/min. All processes were performed under nitrogen atmosphere.

*XRD*. (X-ray diffraction spectrometry) XRD curves were performed on a DX-1000 X-ray diffract meter (Dangdong Fangyuan Instrument Co., Ltd., Liaoning, China) using Cu K*a* radiation (λ = 0.154 nm). The instrument was operated at 40 kV and 20 mA. The sample was measured from 2θ = 5° to 35° at the scanning rate of 2°/min.

*POM*. Morphology and growth rate of spherulite during the isothermal crystallization was observed by a polarizing optical microscopy (POM) (Axio Scope 1, Carl Zeiss, Oberkochen, Germany) equipped with hot stage. Samples were first melted to 240 °C for 4 min to eliminate heat history, and then cooled to the certain temperature (160–180 °C) at 50 °C/min to observe spherulite growth. The size of the growing spherulite was measured by acquiring an image at an appropriate time interval. The average growth rate of the spherulite (*G*) was acquired from the slope of the plot of the spherulite radius versus growth time.

## 3. Results and Discussion

### 3.1. Effect of L/D Ratios on Stereocomplexation of PLLA/PDLA-PEG-PDLA Blends

**Figure 1:**
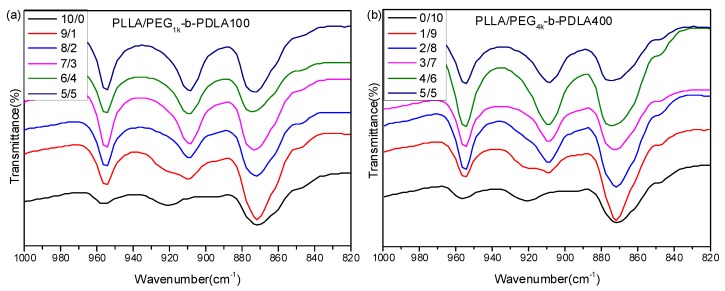
Fourier transform infrared spectrometry (FT-IR) spectra of PLLA/PDLA-PEG_1k_-PDLA100 (**a**) and PLLA/PDLA-PEG_4k_-PDLA400 (**b**) with different L/D ratios.

Figure 1 shows FT-IR spectra of PLLA and the blends with different L/D ratios. For the neat PLLA, there is an obvious absorption peak at 921 cm^−1^, which is a characteristic peak of α-form homocrystallites with a 10_3_ helical chain conformation [34]. It is clear that a novel absorption peak at 908 cm^−1^ is observed as the L/D ratios decreases. Also, the peak at 921 cm^−1^ disappears when the L/D ratio is smaller than 7/3. This peak at 908 cm^−1^ is the characteristic peak of stereocomplex crystallites, which is attributed to the combination of the CH_3_ rocking vibration and the C–COO stretching vibration modes of molecular chains with the 3_1_ helical conformation in the sc-crystalline phase [35]. This reveals that the full stereocomplex PLA could be formed in the blends of PLLA/PDLA-PEG-PDLA when the L/D ratio is smaller than 7/3.

**Figure 2:**
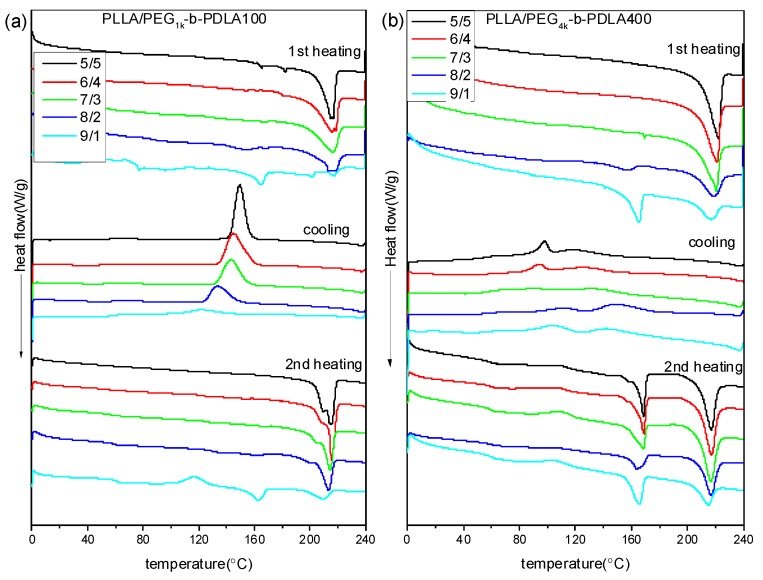
Differential scanning calorimetry (DSC) curves of PLLA/PDLA-PEG_1k_-PDLA100 (**a**) and PLLA/PDLA-PEG_4k_-PDLA400 (**b**) with different L/D ratios.

Figure 2 shows DSC curves of PLLA/PDLA-PEG-PDLA blends with different L/D ratios. DSC curves of the neat PLLA were also measured, as shown in Figure S2. The melting temperature of the neat PLLA in the first heating scan is about 168 °C and no crystallization peak is observed during cooling. In the second heating scan, the presented exothermic peak at 113 °C is related to the cold crystallization peak, while the two endothermic peaks at 162 and 168 °C are assigned to the melting of PLLA. DSC curves of PLLA/PDLA-PEG_1k_-PDLA100 blends with different L/D ratios are displayed in Figure 2a. In the first heating scan, the melting peak of PLLA decreases as the ratios of L/D decreases, while a novel melting peak of sc-crystallites at about 220 °C is observed. This is because the PLLA chain and the PDLA block in the PDLA-PEG-PDLA could form sterecomplex crystallites through strong hydrogen bonding, which is different from homocrystallites. Also, the melting peak of the homocrystallites disappears when the L/D ratios approach 7/3, indicating that only sc-crystallites are formed in the PLLA/PDLA-PEG_1k_-PDLA100 blends. This is consistent with the FT-IR result. In the cooling process, an obvious exothermic peak appears, which is assigned to melting crystallization of the blends. Additionally, the temperature corresponded to crystallization peak shifts from 121.0 to 149.3 °C with decreasing L/D ratios from 9/1 to 5/5. The enthalpy of the crystallization peak also increases. In the second heating scan, the DSC curve of the blends with 9/1 of L/D ratio show an exothermic peak at about 115 °C, attributed to cold crystallization of PLLA. The cold crystallization peak first decreases and eventually disappears as the L/D ratio decreases. It is also observed that the melting peak of the homocrystallites disappears when the L/D ratios approach 7/3. The melting peak of sc-crystallites presents multi-melting peaks when the L/D ratio is below 7/3, indicating a melting-crystallization-remelting process. This is closely related to the perfect degree of sc-crystallites. These results indicate that the full stereocomplex PLA could be obtained in the PLLA/PDLA-PEG_1k_-PDLA100 blends, which is not related to the prepared method of PLA stereocomplex (solution blending or melt-blending).

It is known that the formation of PLA stereocomplex is affected by the length of the polymer chains [36,37]. Therefore, to analyze in depth the formation mechanism of the stereocomplex between PLLA and PDLA-PEG-PDLA, PLLA/PDLA-PEG_4k_-PDLA400 blends that have similar composition to PLLA/PDLA-PEG_1k_-PDLA100 were also prepared. DSC curves of PLLA/PDLA-PEG_4k_-PDLA400 blends with different L/D ratios are shown in Figure 2b. The first heating scan shows a similar phenomenon with the blends PLLA/PDLA-PEG_1k_-PDLA100 with different L/D ratios, indicating that the PLLA/PDLA-PEG_4k_-PDLA400 blends could also achieve the full stereocomplex by solution blending when the L/D ratios are lower than 7/3. However, DSC curves of the blends PLLA/PDLA-PEG_4k_-PDLA400 are different to PLLA/PDLA-PEG_1k_-PDLA100 during the cooling and reheating process. In the cooling process, there are two exothermic peaks. Two melting peaks are observed at about 165 and 216 °C in the corresponding reheating scan, indicating the formation of homocrystallites and sc-crystallites in the PLLA/PDLA-PEG_4k_-PDLA400 blends. This indicates that it is difficult for full stereocomplex formation in the PLLA/PDLA-PEG_4k_-PDLA400 blends by melt-blending. Because the viscosity during the melt-blending process is higher than that of solution blending the polymer chains could not be fully stretched. Although PDLA-PEG_1k_-PDLA100 and PEG_4k_-b-PDLA400 have similar composition, the chain length of PDLA-PEG_4k_-PDLA400 is longer than that of PEG_1k_-*b*-PDLA100. So the mobility capacity of PDLA-PEG_4k_-PDLA400 is poor with respect to that of PDLA-PEG_1k_-PDLA100, which would decrease the possibility of stereocomplexation between PLLA and PDLA-PEG-PDLA. The temperature attributed to crystallization peaks in Figure 2b first increases and then decreases as the L/D ratio decreases. This may be attributed to PEG block in the blends. Our previous investigation demonstrated that PEG has two sides with respect to PLA crystallization: accelerating the mobility of the polymer chains and suppression of the nucleation [25]. The decreasing L/D ratio results in increasing PEG content in the blends. This affects the interaction between the PLLA chain and PDLA chain, leading to difficulty of nucleation.

**Figure 3:**
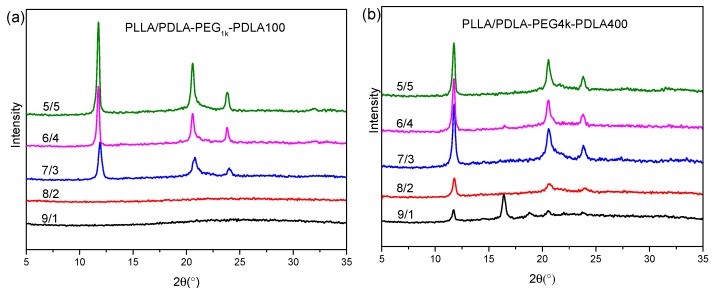
X-ray diffraction (XRD) curves of PLLA/PDLA-PEG_1k_-PDLA100 (**a**) and PLLA/PDLA-PEG_4k_-PDLA400 (**b**) with different L/D ratios (Each sample was first melted at 240 °C for 4 min and then cooled to room temperature at 10 °C/min).

Figure 3 shows XRD curves of the blends with L/D ratios after treatment using the same thermal program as Figure 2. As shown in Figure 3a, XRD curves of the PLLA/PDLA-PEG_1k_-PDLA100 blends with high L/D ratios (≥8/2) do not present any diffraction peaks, while their DSC cooling curves show a crystallization peak (as shown in Figure 2a). This may be related to the crystallinity and sensitivity of the XRD instrument. With increasing D/L ratios, several obvious diffraction peaks are shown at 11.9°, 20.6°, and 23.8°, assigned to the (110), (300)/(030) and (220) planes of sc-crystallites, respectively. They are the characteristic diffraction peaks of sc-crystallites [36,38]. The height of the diffraction peaks increase as the L/D ratios decrease, indicating the formation of more perfect crystallites. Figure 3b shows XRD curves of PLLA/PDLA-PEG_4k_-PDLA400 with different L/D ratios. For the blend with 9/1 of L/D ratio, not only the diffraction peaks of sc-crystallites but also those at 16.3° and 18.6° corresponding to the (110)/(220) and (203) planes of hc-crystallites are observed [36,38]. The diffraction peaks of sc-crystallites strengthen and the diffraction peaks of hc-crystallites disappear as the L/D ratios decrease. However, DSC cooling curves of PLLA/PDLA-PEG_4k_-PDLA400 in Figure 2b show double crystallization peaks, assigned to the crystallization of sc-crystallites and hc-crystallites, respectively. During the cooling, sc-crystallites are first formed and grow. With further decreasing temperature, hc-crystallites are formed due to homogenous nucleation sites formed by the decreased mobility of the polymer chains or heterogeneous nucleation sites of the formed sc-crystallites. However, the growth of hc-crystallites is limited due to the cross-linking effect of the sc-crystallites formed in the blends [39,40]. Therefore, XRD measurement is not sensitive enough for hc-crystallites due to the small size of crystal. In contrast to Figure 3a,b, it can also be concluded that crystallization capacity of PLLA/PDLA-PEG-PDLA blends is related to the PEG blocks, and the longer PEG block shows stronger accelerating crystallization capacity than that of the lower PEG block.

**Figure 4:**
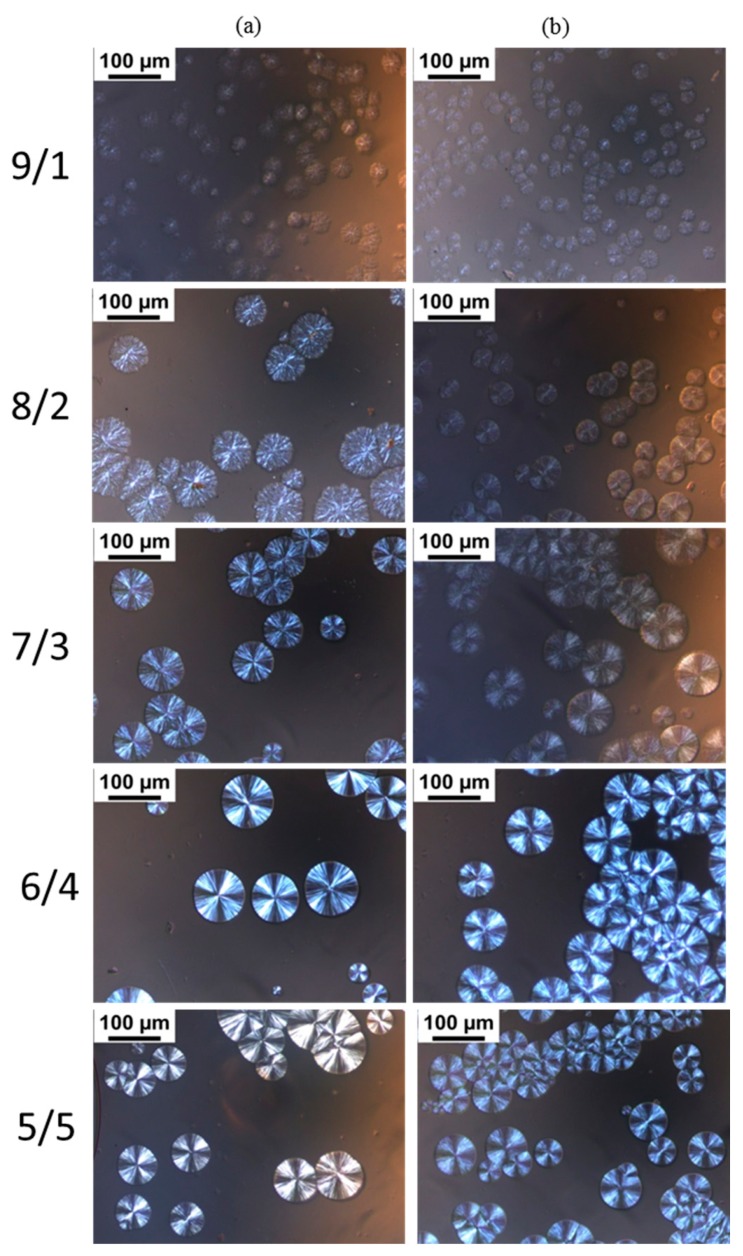
Polarizing optical microscopy (POM) images of the blends with different L/D ratios isothermally crystallized at 160 °C: (**a**) PLLA/PDLA-PEG_1k_-PDLA100; (**b**) PLLA/PDLA-PEG_4k_-PDLA400.

POM images of the blends with different L/D ratios isothermally crystallized at 160 °C are shown in Figure 4. The previous literature references reported that neat PLLA during isothermal crystallization forms spherulites with typical Maltese cross patterns [41,42,43], and it is difficult for PLLA to crystallize at 160 °C, approaching the melting temperature of PLLA [44]. However, we found in our experiments that PDLA-PEG-PDLA could obviously promote PLLA crystallization at 160 °C, assigned to the formation of sc-crystallites and the accelerating effect of PEG for mobility of the polymer chains. For the blends with L/D ratio of 9/1, there are larges of the disordered spherulites and the spherulites are not typical Maltese cross patterns. With decreasing L/D ratios, the spherulites become ordered and present typical Maltese cross patterns. When the L/D ratio is lower than 7/3, the L/D ratio has no significant effect on the morphology of the spherulites. The disordered spherulites could be ascribed to the following: (i) the presence of an excess amount of PLLA which could not take part in stereocomplex formation and either excess enantiomeric polymer could have disturbed the orientation of the stereocomplex lamellae [45]; (ii) the molten amorphous PEG also affects the orientation of the stereocomplex lamellae. It can also be observed that for PLLA/PEG_4k_-*b*-PDLA400 with lower L/D ratios (≤7/3), the contrast between the bright and dark regions of the Maltese crosses is unclear with respect to PLLA/PDLA-PEG_1k_-PDLA100 with similar L/D ratios. This may be attributable to the decreased orientation of lamellae because the entanglement increases as the polymer chain extends [46].

### 3.2. Effect of PEG Block on Stereocomplexation of PLLA/PDLA-PEG-PDLA Blends

**Figure 5:**
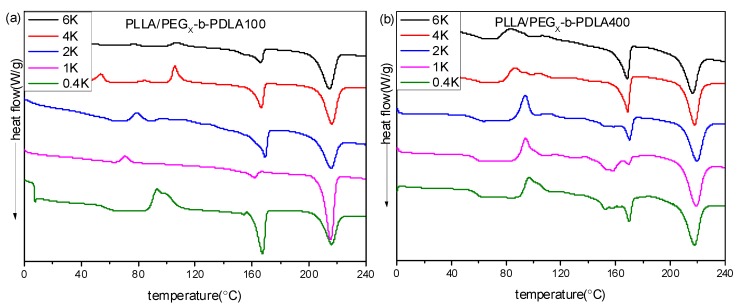
DSC heating curves of blends with different PEG blocks after quenching from 240 °C: (**a**) PLLA/PDLA-PEG_1k_-PDLA100; (**b**) PLLA/PDLA-PEG_4k_-PDLA400.

Figure 5a shows DSC heating curves of PLLA/PDLA-PEG_X_-PDLA100 blends with different PEG blocks after quenching from 240 °C. For PLLA/PDLA-PEG_0.4k_-PDLA100, there is a double exothermic peak at 93.5 and 100.2 °C, assigned to cold crystallization of sc-crystallines and hc-crystallines, respectively. There only appeared a cold crystallization peak with further increasing PEG blocks, and the temperature corresponded to cold crystallization shifts to lower temperature. When the PEG block approaches or exceeds 2 kg/mol, multiple cold crystallization peaks are observed. From Figure S3, the cold crystallization temperatures of the neat PLLA and PDLA-PEG_4k_-PDLA100 are 113.5 and 117.3 °C, respectively. Therefore, the cold crystallization peak at about 105 °C may be attributed to the crystallization of hc-crystallites of PDLA-PEG-PDLA or PLLA. The cold crystallization at the lower temperature region is attributed to the crystallization of sc-crystallites in the blend. As shown in Figure 5a, the blends show two melting peaks at 160 and 216 °C, assigned to the melting of hc-crystallites and sc-crystallites, respectively. It is clear that the ratio of the peak areas assigned to sc-crystallites and hc-crystallites first increases and then decreases with increasing PEG block, and this ratio is maximum when the PEG block is 1.0 kg/moL.

DSC heating curves of PLLA/PDLA-PEG_X_-PDLA400 blends with different PEG blocks after quenching from 240 °C are shown in Figure 5b. All blends show an obvious step at about 50–57 °C, assigned to the glass transition of PLA. The glass transition temperature shifts to lower temperature with increasing PEG block, which is because PEG has a lower glass transition temperature (−60 °C) [47]. For PLLA/PDLA-PEG_0.4k_-PDLA400, its cold crystallization peak appears at about 96.7 °C. It can be seen that the temperature corresponding to cold crystallization shifts to the lower temperature region with increasing PEG blocks. It is clear from Figure S3 that the cold crystallization temperatures of the neat PLLA and PDLA-PEG_4k_-PDLA400 are 113.5 and 81.9 °C, respectively. This reveals that the PEG block of the blends not only accelerates the arrangement of PDLA-PEG-PDLA chains, but is also favorable for the mobility of PLLA. Therefore, the cold crystallization peak shifts to the lower temperature region with increasing PEG blocks. The PLLA/PDLA-PEG_0.4k_-PDLA400 blend displays a multiple endothermic peak at 140–175 °C, assigned to the melting of PLA hc-crystallines. With increasing the PEG block, the melting peak of the hc-crystallites becomes narrow, indicating that the increased PEG blocks obviously promote the crystallization of PLA hc-crystallines, and improve the degree of perfect PLA hc-crystallines. The endothermic peak at about 220 °C is assigned to the melting of the sc-crystallites. Also, the area of this peak corresponding to sc-crystallites decreases with increasing PEG block. The results indicate that the PEG block is not beneficial to the formation of sc-crystallites in the PLLA/PDLA-PEG_X_-PDLA400 blends. The above findings reveal that the PEG block plays a different role in the PLLA/PDLA-PEG_X_-PDLA100 blends and PLLA/PDLA-PEG_X_-PDLA400 blends. For the blends with long PDLA, it is difficult for an arrangement of polymer chains in the stereocomplex. Although the PEG block could accelerate the arrangement alternately of PDLA-PEG-PDLA and PLLA to form sc-crystallites, it could also promote the single arrangement of PDLA-PEG-PDLA or PLLA to form hc-crystallites. As shown in Figure 5b, the longer PEG block presents an obvious accelerating effect for the formation of hc-crystallites, which is attributed to the aid of an accelerator for chain mobility. Therefore, the above results reveal that the accelerating effect of PEG blocks for PLLA/PDLA-PEG_X_-PDLA100 is obvious with respect to PLLA/PDLA-PEG_X_-PDLA400.

**Figure 6:**
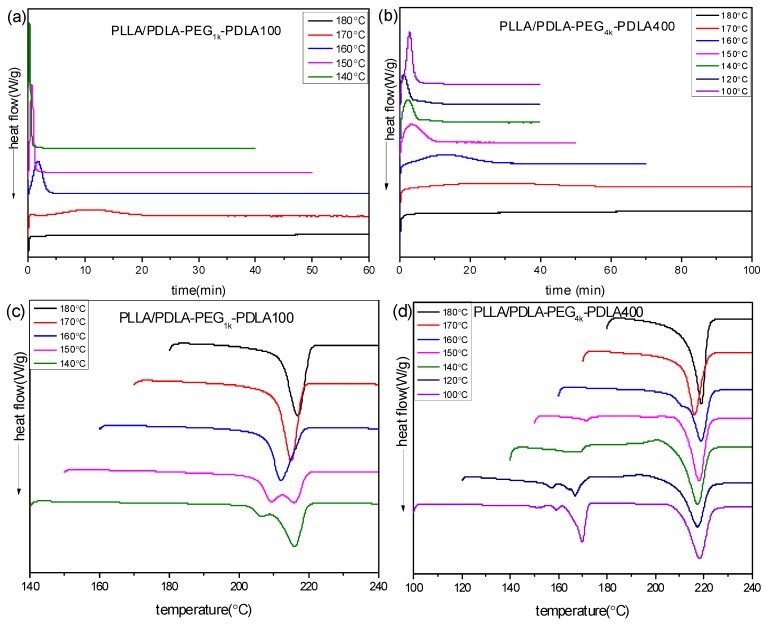
DSC curves of isothermal crystallization process (**a**,**b**) at different temperatures and subsequently heating processes (**c**,**d**): (**a**,**c**) PLLA/PDLA-PEG_1k_-PDLA100 and (**b**,**d**) PLLA/PDLA-PEG_4k_-PDLA400.

To further investigate the effect of PEG blocks on crystallization of PLLA/PDLA-PEG-PDLA blends, the isothermal crystallization behavior of PLLA/PDLA-PEG_X_-PDLA100 and PLLA/PDLA-PEG_X_-PDLA400 was measured. Figure 6 shows the DSC curves of PLLA/PDLA-PEG_1k_-PDLA100 and PLLA/PDLA-PEG_4k_-PDLA400 isothermal-crystallized at different temperatures, and the sequent DSC re-heating curves. As shown in Figure 6a, the isothermal crystallization curve of PLLA/PDLA-PEG_1k_-PDLA100 at a given temperature shows only an exothermic peak, and the time corresponding to this peak shifts to a shorter time region with decreasing of *T*_c_ in the temperature range of 140–180 °C. We found that a sharp and fast exothermic peak presents during the cooling process with a cooling rate of 50 °C/min at lower *T*_c_ (<140 °C). So the crystallization peak at lower *T*_c_ could not be recorded. This is related to the fast crystallization capacity of PLLA/PDLA-PEG_1k_-PDLA100. In Figure 6b, a similar phenomenon with Figure 6a presents in that the crystallization peak of PLLA/PDLA-PEG_4k_-PDLA400 shifts to a shorter time region with decreasing *T*_c_ at 140–180 °C. However, the crystallization peak shifts to a longer time region on further decreasing *T*_c_ (<140 °C). This is attributed to the difficulty of nucleation and chain diffusion at higher *T*_c_ and lower *T*_c_, respectively. An analogous phenomenon was reported by Pan and Tsuji [35,13]. These results indicate that the crystallization capacity of PLLA/PDLA-PEG_1k_-PDLA100 is stronger than that of PLLA/PDLA-PEG_4k_-PDLA400. Although PDLA-PEG_1k_-PDLA100 and PDLA-PEG_4k_-PDLA400 have a similar composition, they have a different block length, which directly affects the segment mobility and arrangement during the crystallization process.

Figure 6c,d show the corresponding melting curves for PLLA/PDLA-PEG-PDLA after isothermal crystallization at different temperatures. PLLA/PDLA-PEG_1k_-PDLA100 crystallized at 140 °C displays a double melting peak at 205 and 215.8 °C, indicating that the melting-recrystallization-remelting process of sc-crystallite occurs there. This is closely related to the perfectness and recrystallizability of this blend. On increasing *T*_c_, the two peaks merge into a novel peak and the temperature assigned to the peak shifts to higher temperature. This reveals that the perfect degree of sc-crystallites increases and its recrystallizability decreases as *T*_c_ increases. However, PLLA/PDLA-PEG_4k_-PDLA400 shows two melting regions at 150–180 °C and 210–230 °C, assigned to homocrystallites and sc-crystallites, respectively. It is clear that hc-crystallites experience a multi-melting process. This is because for hc-crystallite crystallization, there are two nucleation mechanisms: (i) heterogeneous nucleation of sc-crystallites; (ii) homogenous nucleation of PLLA or PDLA-PEG-PDLA. Eventually, this leads to the noticeable difference of the crystallites. The melting peak of hc-crystallites decreases as the *T*_c_ increases. Eventually, the melting peak of hc-crystallites disappears when *T*_c_ is higher than 150 °C. Figure 6d shows an obvious exothermic peak in front of the sc-crystallite melting with increasing *T*_c_. On further increasing *T*_c_, this exothermic peak decreases and even disappears when *T*_c_ is higher than 150 °C. However, the melting peak is split into two peaks at *T*_c_ = 160 °C. At higher *T*_c_ (>170 °C), the two peaks are merged into one peak and the temperature corresponding to this peak shifts to a higher temperature region. These results reveal that PLLA/PDLA-PEG_4k_-PDLA400 isothermally crystallized at lower *T*_c_ forms crystallites poorly perfect due to the co-crystallization of hc-crystallite and sc-crystallite. With increasing *T*_c_, the formation of hc-crystallites is hindered and the polymer segment presents a stronger mobility capacity, which is favorable for the formation of sc-crystallites of high perfection. In contrast to Figure 6c,d, it can be found that the melting temperature of sc-crystallites isothermally crystallized in the PLLA/PDLA-PEG_4k_-PDLA400 blend is higher than that of PLLA/PDLA-PEG_1k_-PDLA100 at the same *T*_c_. The long PDLA block in the PLLA/PDLA-PEG-PDLA blends is beneficial to the formation of thick lamellae.

To further investigate the effect of the PEG block on the crystallization behavior of PLLA/PDLA-PEG-PDLA, the isothermal crystallization behavior of PLLA/PDLA-PEG_X_-PDLA100 and PLLA/PDLA-PEG_X_-PDLA400 at 160 °C was also measured, and the obtained curves are shown in Figure S4, which was analyzed by the Avrami equation [48,49]:

$$X_{t}=\frac{\int_{0}^{t}(dH/dt)dt}{\int_{0}^{\infty }(dH/dt)dt}$$

where *X*_t_ is the relative degree of crystallinity; *k* is the crystallization rate constant; *t* is the crystallization time; and the value of *n* represents the Avrami exponent correlating with the nucleation mechanism and crystal growth dimension [49,50]. In order to ensure the accuracy of Avrami plotting, only the data within *X*_t_ = 5%–50% was used in the analysis. The plot of log[–ln(1–*X*_t_)] versus log*t* obtained from Figure S5 is a straight line, and the obtained data are listed in Table 2. The *n* values of PLLA/PDLA-PEG_X_-PDLA100 and PLLA/PDLA-PEG_X_-PDLA400 are around 2.50–3.13 and 2.08–3.65, respectively. This reveals a three-dimensional crystallization growth and homogenous nucleation mechanism, and the two blends exhibits different self-nucleated capacity. It is clear that the *n* values are independent of the PEG block but the *k* value first decreases and then increases with increasing PEG block. Short PEG block obviously prefers decreased nucleation density with respect to PLLA/PDLA, while long PEG block is mainly favorable for the accelerating effect for chain mobility. It can also be found that the *k* value of PLLA/PDLA-PEG_X_-*b*-PDLA100 is higher than that of PLLA/PDLA-PEG_X_-b-PDLA400 with similar PEG block, indicating that PEG presents the obvious accelerating crystallization capacity for the blends with short PDLA block. Half-time of crystallization (*t*_1/2_) is defined as the time spent from the onset of crystallization to the point with *X*_t_ = 50%. The *t*_1/2_ values of PLLA/PDLA-PEG_X_-PDLA100 and PLLA/PDLA-PEG_X_-PDLA400 isothermally crystallized at 160 °C are listed in Table 2. For PLLA/PDLA-PEG_X_-PDLA100, *t*_1/2_ shows a minimum (1.76 min) for the blend with PEG block of 1 kg/mol. The minimum *t*_1/2_ value of PLLA/PDLA-PEG_X_-PDLA400 is 5.66 min at PEG block of 6 kg/mol and the *t*_1/2_ value is not dependent on the PEG block. The crystallization rate can be easily described by the reciprocal of *t*_1/2_, which is affected by these factors: nucleation and crystal growth. At a certain range of PEG block, PEG plays the role of accelerating agent for the mobility of polymer chains and does not affect the nucleation. When PEG block exceeds a certain range, PEG shows the effect of decreasing the nucleation capacity due to its diluting effect, while keeping its effect to accelerate the mobility of the polymer chains. Also, the fast movement capacity of the polymer chains also presents two sides to the crystallization the of PLLA/PDLA-PEG-PDLA blends. Therefore, the problem of the effect of PEG block on PLLA/PDLA-PEG-PDLA blends will be further discussed in the future.

The spherulite morphology and growth rate of PLLA/PDLA-PEG_X_-PDLA100 and PLLA/PDLA-PEG_X_-PDLA400 isothermally crystallized at 170 °C were observed by POM. As shown in Figure S6, all blends form spherulites with typical Maltese cross patterns. The plot of *G* versus time calculated from POM is displayed in Table 2. The radial growth rate of spherulites follows basically the trend: it first increases and then decreases with increasing PEG block. This is related closely to the accelerating mobility capacity of PEG block for polymer chains. With short PEG block, the PEG block in the blends presents a suitable accelerating capacity for the mobility of the polymer chains, which is helpful to the arrangement of polymer chains to form a crystal. However, longer PEG block in the blends offers a fast movement capacity for the polymer chains, which is a disadvantage for the polymer chains arrangement in the lattice. Additionally, the longer PEG block leads to difficulty of nucleation. Song et al. [24] demonstrated that the diluting effect by residual PEG in PLLA/PDLA-*b*-PEG-*b*-PDLA blends might result in a decrease of nucleation density. It can be observed that the *G* value of PLLA/PDLA-PEG_X_-PDLA400 is higher than that of PLLA/PDLA-PEG_X_-PDLA100, assigned to the strong hydrogen bond between enantiomer chains.

### 3.3. Effect of PDLA Block on Stereocomplexation of PLLA/PDLA-PEG-PDLA Blends

**Figure 7:**
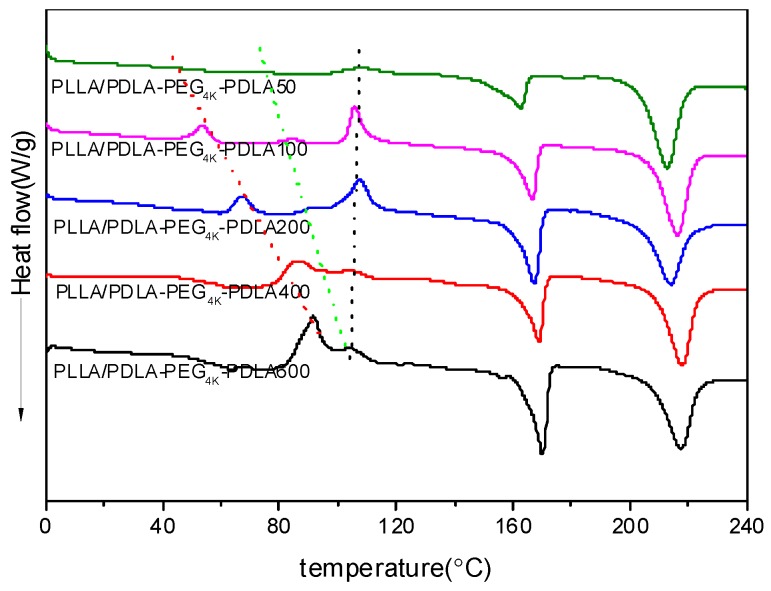
DSC heating curves of PLLA/PDLA-PEG_4k_-PDLA blends with different PDLA blocks after quenching from 240 °C.

Figure 7 shows DSC heating curves of PLLA/PDLA-PEG_4k_-PDLA Y blends with different PDLA blocks after quenching from 240 °C. For PLLA/PDLA-PEG_4k_-PDLA50, a cold crystallization peak shows at 107.6 °C. However, the low temperature region shows two novel cold crystallization peaks and the temperatures assigned to these peaks shift to the higher temperature region as the PDLA block increases. When the length of PDLA block reaches 600 in PLLA/PDLA-PEG_4k_-PDLA, the several cold crystallization peaks begin to coincide. The possible reason is because the increasing the PDLA block decreases the mobility capacity of the polymer chains. The cold crystallization temperatures of the neat PLLA and PDLA-PEG_4k_-PDLA are shown in Figure S3. Therefore, the three cold crystallization peaks at the lowest temperature regions, intermediate and highest temperature regions can be attributed to the crystallization of sc-crystallites, PDLA-PEG-PDLA and PLLA, respectively. All blends show two endothermic peaks at about 170 and 230 °C, assigned to hc-crystallites and sc-crystallites, respectively. For PLLA/PDLA-PEG_4k_-PDLA50, the area attributed to the melting of hc-crystallites is larger than the area of the cold crystallization peak, indicating that hc-crystallites are also formed during the cooling process at 50 °C/min. This may be attributed to the heterogeneous nucleation of the formed sc-crystallites and the accelerating effect of PEG on the mobility of the polymer chain. Furthermore, the melting peak area of hc-crystallites increases with increasing PDLA block. This is because *t* the increasing PDLA block decreases the mobility capacity of the polymer segments, affecting the accumulation of PLLA segments and PDLA segments in the blends by the strong van der Waals forces of CH_3_^…^C=O interactions [12]. This is favorable for PLLA or PDLA-PEG-PDLA to form hc-crystallites. Again, the existed obvious competitive relationship between sc-crystallites and hc-crystallites in the PLLA/PDLA-PEG_4k_-PDLA is strongly related to the different length of PDLA blocks, which is a disadvantage for the formation of sc-crystallites as the PDLA block increases.

**Figure 8:**
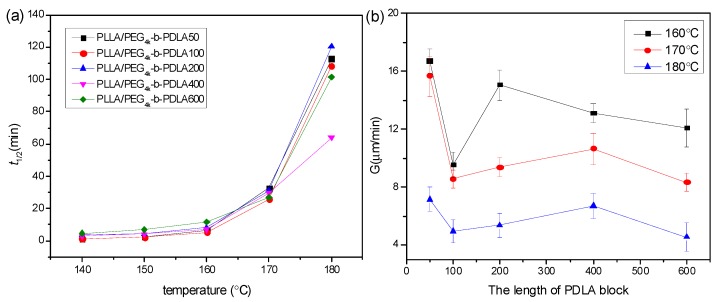
(**a**) Crystallization half-time (*t*_1/2_) and (**b**) radial growth rate of spherulites of (*G*) of PLLA/PDLA-PEG_4k_-PDLA with different PDLA blocks isothermally crystallized at different temperatures.

**Figure 9:**
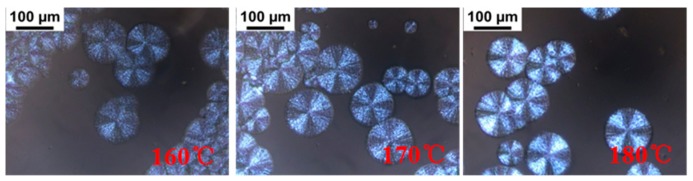
POM images of PLLA/PDLA-PEG_4k_-PDLA600 isothermally crystallized at different temperatures.

The isothermal crystallization kinetics of PLLA/PDLA-PEG_4k_-PDLA Y with different PDLA blocks were further studied using DSC at the temperature ranges of 140–180 °C, and the measured curves of heat flow versus time are displayed in Figure S7. The obtained curves of *X*_t_ versus *t* (Figure S8) were investigated according to the Avrami equation, and the plots of log[–ln(1–*X*_t_)] versus log*t* are shown in Figure S9 and the data obtained from Figure S9 are listed in Table S1. The *n* values of the blends with different PDLA blocks vary from 2.06 to 3.39, and this parameter is independent of the PDLA blocks. It is clear that the crystallization rate constant *k* decreases with increasing *T*_c_, which is because higher *T*_c_ results in difficulty of nucleation. It is observed that the *k* value first increases and then decreases as the PDLA block extends. At short PDLA block, the increase of PDLA is helpful to nucleation, while the mobility of the polymer chains is limited at long PDLA block. Figure 8a shows the crystallization half-time of PLLA/PDLA-PEG_4k_-PDLAY isothermally crystallized at a temperature of 140–180 °C. The values of *t*_1/2_ increases as *T*_c_ increases, which is unfavorable for nucleation. The *t*_1/2_ remains almost a constant value when the PDLA block is lower than 200. When the PDLA block exceeds 200, the *t*_1/2_ first decreases and then increases. This may be attributed to the difficulty of nucleation and chain diffusion at short and long PDLA block, respectively. The previous literature found that residual PEG was a disadvantage for PLLA nucleation [17,28]. The increase of PDLA block promotes nucleation, but the mobility of the polymer segment is limited due to the winding between polymer chains. The spherulite morphology and growth rate of PLLA/PDLA-PEG_4k_-PDLAY were also observed by POM. As shown in Figure S10, the blends show typical Maltese cross patterns at the temperature of 160–180 °C when the PDLA block varies from 50 to 400. However, the blend PLLA/PDLA-PEG_4k_-PDLA600 displays a different morphology, as shown in Figure 9. Although the spherulites of PLLA/PDLA-PEG_4k_-PDLA600 present typical Maltese cross patterns, their surfaces show a similar structure with walnut. This disordered structure could be assigned to the decreased orientation of the lamellae. This is because the increase of PDLA block results in the increase of entanglements among the polymer segments [42]. Growth rate of spherulites (*G*) is also calculated as shown in Figure 8b. The *G* value decreases with increasing *T*_c_. The higher *T*_c_ is a disadvantage for the arrangement of the polymer chains and with increasing PDLA block, the *G* value obviously changes. The increase of PDLA block could decrease the movement capacity of the polymer chains, and could increase the hydrogen bonding effect between the PLLA chain and the PDLA chain as well as the winding between the polymer chains.

**Figure 10:**
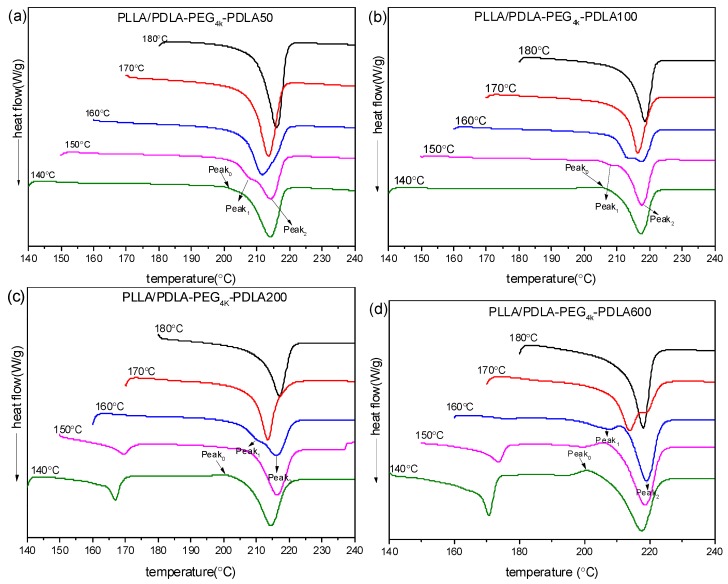
DSC heating curves of PLLA/PDLA-PEG_4k_-PDLA with different PDLA blocks isothermally crystallized at different temperatures: (**a**) PLLA/PDLA-PEG_4k_-PDLA50; (**b**) PLLA/PDLA-PEG_4k_-PDLA100; (**c**) PLLA/PDLA-PEG_4k_-PDLA200; (**d**) PLLA/PDLA-PEG_4k_-PDLA600.

The subsequent DSC heating curves are shown in Figure 10. It is clear that PLLA/PDLA-PEG_4k_-PDLA50 shows only a melting peak at about 220 °C under all *T*_c_ investigated, indicative of the exclusive formation of sc-crystallites. It can be found that a weak exothermic peak (Peak_0_) presents prior to the melting peak. This exothermic peak gradually disappears and a melting endotherm appears at lower temperature (Peak_1_) prior to an endotherm at higher temperature (Peak_2_), with increasing *T*_c_. Peak_1_ may be attributed to the melting of primary crystals developed in the isothermal crystallization and Peak_2_ is assigned to the melting of sc-crystallites formed in recrystallization upon heating. It is notable that Peak_1_ gradually shifts to higher temperature and Peak_2_ shifts to lower temperature as *T*_c_ increases. Eventually, Peak_1_ and Peak_2_ merge into a novel peak, and this peak shifts to higher temperature as *T*_c_ increases. This is because the more perfect crystallites with higher melting temperature are formed and the fraction of crystallites undergoing recrystallization decreases with increasing *T*_c_. A similar phenomenon was reported by Pan et al. [35]. It is also clear that a novel exothermic peak at about 160–170 °C at lower *T*_c_ (≤150 °C) with increasing PDLA block, is assigned to melting of hc-crystallites. The area of the peak corresponding to hc-crystallites increases at similar *T*_c_ as the PDLA block extends, which is consistent with the results of non-isothermal crystallization. This is because the increase of PDLA block decreases the suppression of PEG block on nucleation. This would promote nucleation site formation of hc-crystallites, which possess poor nucleation capacity with respect to sc-crystallites. Therefore, PEG block tends to favor the creation of sc-crystallites and the increase of PDLA block progressively annihilates this effect.

## 4. Conclusions

In this study, PLA stereocomplex was investigated based on the blending between PLLA and PDLA-PEG-PDLA copolymer, in which PEG soft segment was introduced in the molecular chain of PDLA. Firstly, the influence of D/L ratios on the stereocomplex degree and crystal morphology of PDLA-PEG-PDLA/PLLA blend was discussed. Full stereocomplexation can be achieved with D/L ratios ranging widely from 3/7–5/5 through solution casting methods due to the soft segment PEG block in the copolymer. However, further study indicated that the stereocomplex degree of the blends during the melting blending was strongly related to the PEG and PDLA blocks. Short PEG and PDLA blocks could achieve the full stereocomplex when D/L ratios are 3/7–5/5.

The effect of PEG blocks on the stereocomplexation of PLLA/PDLA-PEG-PDLA with similar composition and different PDLA blocks was studied. The PEG blocks can obviously accelerate the stereocomplexation of the blends with short PDLA blocks. The blends with longer PDLA blocks can achieve the full stereocomplex by increasing the crystallization temperature. The PEG blocks play two roles in PLA stereocomplexation: accelerating mobility of the polymer chains and decreasing nucleation capacity because of their diluting effect.

The effect of PDLA block on the stereocomlexation of the blends was also investigated. The increase of PDLA block in the copolymer was favorable for the formation of hc-crystallites. The competition between the formation of hc-crystallites and sc-crystallites in the PLLA/PDLA-PEG_4k_-PDLA was strongly related to the PDLA segments. Isothermal crystallization revealed that t_1/2_ first decreases and then increases as the PDLA block increases. POM results revealed that the spherulite morphology of PLLA/PDLA-PEG_4k_-PDLA600 isothermally crystallized showed a similar structure to walnut. The influence of the temperature of isothermal crystallization on the PLA stereocomplex was also investigated by isothermal measurements. The melting behavior of PLLA/PDLA-PEG_4k_-PDLA with different PDLA blocks after isothermal crystallization showed that the blends could form a full stereocomplex when the crystallization temperature exceeded 160 °C, and a crystallite with high perfection was formed as the crystallization temperature increased.

To sum up, this study illustrated systemically the effects of the L/D ratios, PEG, PDLA blocks and crystallization conditions on PLA stereocomplex degree, crystalline morphology, and crystallization capacity of PLLA/PDLA-PEG-PDLA blends. This can provide potential approaches to control the microstructure and physical performance of PLLA/PDLA-PEG-PDLA blends, as well as the assembled structures and properties of copolymer systems containing PLLA, PDLA, and PEG blocks.

## Figures and Tables

**Table 1:** Synthesis and characterization of poly(d-lactide)-poly(ethylene glycol)-poly(d-lactide).

Code	*M*_the_^a^ (×10^4^ g/moL)	*M*_n_^b^ (×10^4^ g/moL)	*M*_n_^c^ (×10^4^ g/moL)	PDI ^c^	*T*_c_^d^ (°C)	*T*_m_^d^ (°C)	Yield (wt %)
PDLA-PEG_4k_-PDLA50	1.12	1.48	1.40	1.56	108	165	73.0
PDLA-PEG_4k_-PDLA100	1.84	2.36	1.78	1.50	107	167	90.6
PDLA-PEG_4k_-PDLA200	3.28	2.93	2.11	1.61	105	169	67.4
PDLA-PEG_4k_-PDLA400	6.16	7.23	2.59	1.65	106	170	76.9
PDLA-PEG_4k_-PDLA600	9.04	9.62	3.41	1.51	106	172	57.4
PDLA-PEG_0.4k_-PLA100	1.48	2.07	1.47	1.40	102	170	93.4
PDLA-PEG_1k_-PDLA100	1.54	2.51	1.43	1.63	112	161	55.8
PDLA-PEG_2k_-PDLA100	1.64	2.91	1.57	1.57	105	171	92.4
PDLA-PEG_6k_-PDLA100	2.04	2.52	1.96	1.72	111	168	68.6
PDLA-PEG_0.4k_-PDLA400	5.80	6.20	2.53	1.71	105	172	86.0
PDLA-PEG_1k_-PDLA400	5.86	7.86	2.43	1.52	110	171	89.2
PDLA-PEG_2k_-PDLA400	5.96	7.10	2.34	1.47	109	170	88.2
PDLA-PEG_6k_-PDLA400	6.16	9.71	2.52	1.61	110	173	55.4

PDI, polydispersity index. ^a^ Determined as the theoretical molecular weight of PDLA-PEG-PDLA calculated by the molar ratio of lactide to polyethylene. ^b^ Calculated based on ^1^H nuclear magnetic resonance spectroscopy. ^c^ Determined by gel permeation chromatography using tetrahydrofuran as the eluent at 40 °C. ^d^ Measured by differential scanning calorimetry (Samples were first melted at 180 °C for 4 min, and then cooled to 0 °C and reheated to 180 °C at 10 °C/min under N_2_ atmosphere).

**Table 2:** Avrami kinetic parameters of PLLA/PDLA-PEG_X_-PDLA100 and PLLA/PDLA-PEG_X_-PDLA400 isothermally crystallized at 160 °C and radial growth rate of spherulites of (*G*) of the blends isothermally crystallized at 170 °C.

PEG_X_	PLLA/PDLA-PEG_X_-PDLA100				PLLA/PDLA-PEG_X_-PDLA400			
	*n*	*k* (min^−n^)	*t*_1/2_ (min)	*G* (um/min)	*n*	*k* (min^−n^)	*t*_1/2_ (min)	*G* (um/min)
0.4k	2.50	4.26 × 10^−3^	7.83	5.00	3.65	1.29 × 10^−3^	7.64	7.85
1k	2.61	4.83 × 10^−4^	1.76	9.98	2.49	1.03 × 10^−3^	6.17	7.73
2k	2.80	1.81 × 10^−3^	8.37	8.57	3.15	1.43 × 10^−3^	7.22	16.10
4k	3.13	2.39 × 10^−3^	5.36	5.36	2.08	1.56 × 10^−2^	13.77	8.93
6k	2.81	3.79 × 10^−3^	7.69	5.00	3.09	1.34 × 10^−3^	5.66	8.34

## Supplementary Materials

The following are available online at www.mdpi.com/2073-4360/9/3/107, Figure S1: ^1^H NMR (A) and ^13^C NMR (B) spectra of PLLA-PEG_1k_-PLLA100 (a) and PDLA-PEG_4k_-PDLA400 (b), Figure S2: DSC curves of the neat PLLA, Figure S3: DSC heating curves of PLLA, PDLA-PEG_4k_-PDLA100, and PDLA-PEG_4k_-PDLA400 blocks after quenching from 240 °C, Figure S4: Isothermal crystallization curves of the blends with different PEG blocks at 160 °C: (a) PLLA/PDLA-PEG_X_-PDLA100; (b) PLLA/PDLA-PEG_X_-PDLA400, Figure S5: Variation of *X*_t_ with crystallization time *t* of PLLA/PDLA-PEG_X_-b-PDLA100 and PLLA/PDLA-PEG_X_-PDLA400 at different crystallization temperatures, Figure S6: POM images of the blends with different PEG blocks isothermally crystallized at 170 °C: (a) PLLA/PDLA-PEG_X_-PDLA100; (b) PLLA/PDLA-PEG_X_-PDLA400, Figure S7: Isothermal crystallization curves of the blends with different PDLA blocks at different temperatures, Figure S8: Variation of *X*_t_ with crystallization time *t* of the blends with different PDLA blocks at different crystallization temperatures, Figure S9: The plots of log[–ln(1–*X*_t_)] versus log*t* at different crystallization temperatures, Figure S10; POM images of the blends with different PDLA blocks isothermally crystallized at different temperatures, Table S1: Avrami kinetic parameters for the isothermal crystallization of PLLA/PDLA-PEG_4k_-PDLA with different PDLA blocks.
