# Supplementary material for: Competitive Stereocomplexation and Homocrystallization Behaviors in the Poly(lactide) Blends of PLLA and PDLA-PEG-PDLA with Controlled Block Length
Source: Polymers (Basel). 2017 Mar 15;9(3):107. doi: 10.3390/polym9030107 (PMC6432197; doi:10.3390/polym9030107)
Supplement: Supplementary file 1 [file polymers-09-00107-s001.pdf]

Supplementary Materials: Competitive Stereocomplexation and Homocrystallization Behaviors in the Poly(lactide) Blends of PLLA and PDLA-PEG-PDLA with Controlled Block Length

Zhanxin Jing, Xuetao Shi and Guangcheng Zhang

Table S1. Avrami kinetic parameters for the isothermal crystallization of PLLA/PDLA-PEG$_{4k}$-PDLA with different PDLA blocks.

Factors	T_c (°C)	n	k (min^{-n})	R^2
PDLA50	140	2.30	0.587	0.9995
	150	2.46	9.75×10^{-2}	0.9998
	160	3.03	2.48×10^{-3}	0.9982
	170	2.54	1.00×10^{-4}	0.9999
	180	2.68	2.15×10^{-6}	0.9999
PDLA100	140	2.72	0.363	0.9998
	150	2.44	0.117	0.9995
	160	3.13	3.79×10^{-3}	0.9985
	170	3.39	1.14×10^{-5}	0.9988
	180	2.74	2.06×10^{-6}	0.9995
PDLA200	140	2.06	5.61×10^{-2}	0.9971
	150	3.07	2.91×10^{-3}	0.9953
	160	2.96	1.48×10^{-3}	0.9969
	170	3.03	1.04×10^{-5}	0.9985
	180	2.14	2.04×10^{-6}	0.9842
PDLA400	140	2.57	5.22×10^{-2}	0.9975
	150	2.24	2.84×10^{-2}	0.9978
	160	2.76	4.83×10^{-4}	0.9993
	170	2.53	1.32×10^{-4}	0.9988
	180	3.07	2.07×10^{-6}	0.9991
PDLA600	140	2.62	1.32×10^{-2}	0.9975
	150	2.39	7.20×10^{-3}	0.9997
	160	2.38	1.99×10^{-3}	0.9976
	170	2.51	1.83×10^{-4}	0.9991
	180	2.18	2.87×10^{-5}	0.9989

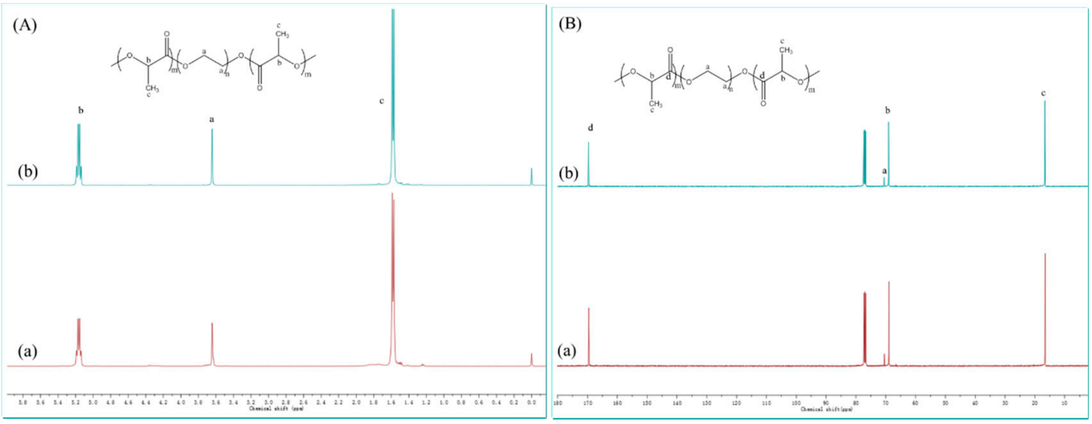

Figure S1. ^{1}H NMR (**A**) and ^{13}C NMR (**B**) spectra of PLLA-PEG$_{1k}$-PLLA100 (**a**) and PDLA-PEG$_{4k}$-PDLA400 (**b**).

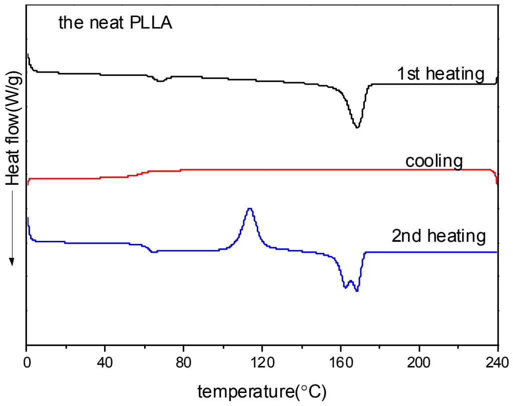

Figure S2. DSC curves of the neat PLLA.

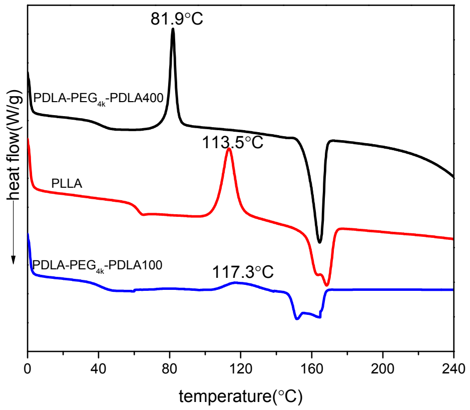

Figure S3. DSC heating curves of PLLA, PDLA-PEG$_{4k}$-PDLA100, and PDLA-PEG$_{4k}$-PDLA400 blocks after quenching from 240 °C.

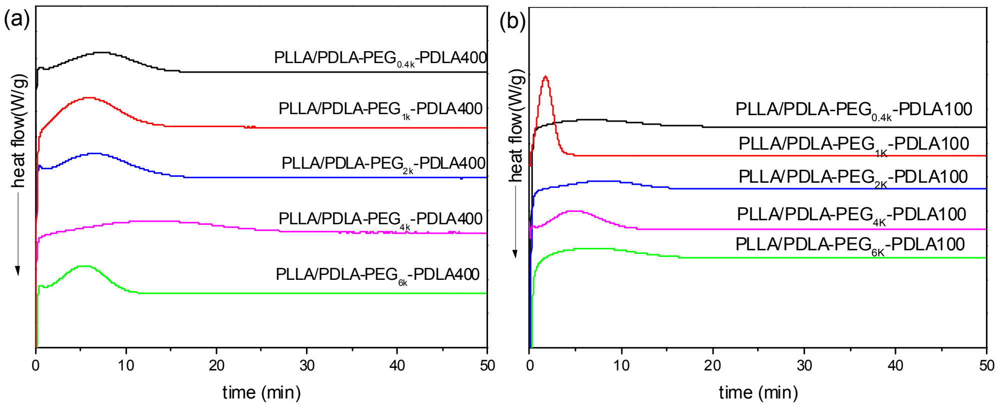

Figure S4. Isothermal crystallization curves of the blends with different PEG blocks at 160°C: (**a**) PLLA/PDLA-PEG$_x$-PDLA100; (**b**) PLLA/PDLA-PEG$_x$-PDLA400.

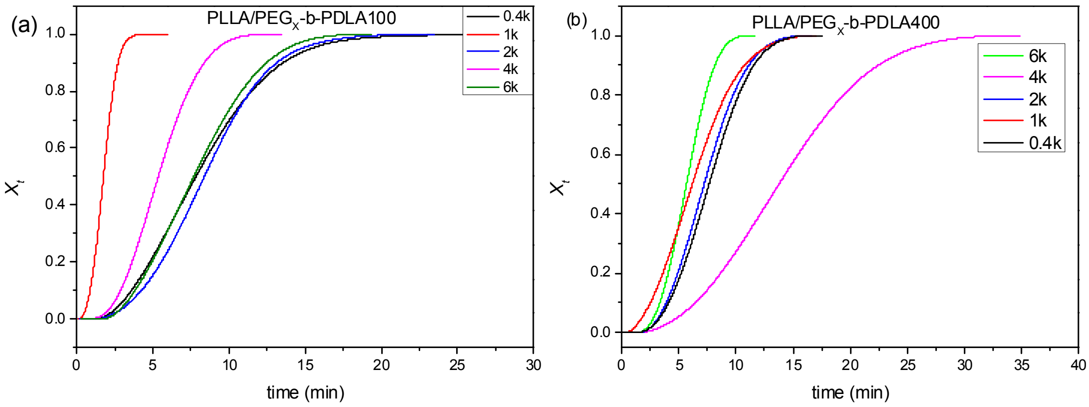

Figure S5. Variation of X_t with crystallization time t of PLLA/PDLA-PEGx-b-PDLA100 and PLLA/PDLA-PEGx-PDLA400 at different crystallization temperatures.

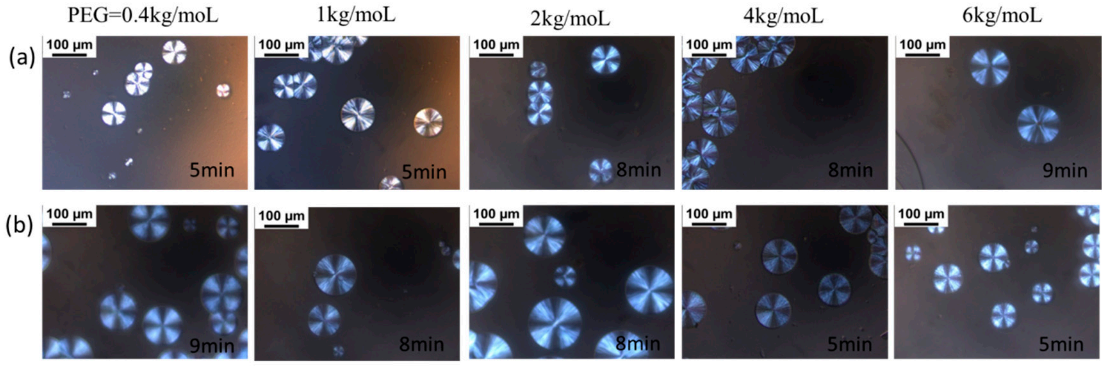

Figure S6. POM images of the blends with different PEG blocks isothermally crystallized at 170 °C: (**a**) PLLA/PDLA-PEGx-PDLA100; (**b**) PLLA/PDLA-PEGx-PDLA400.

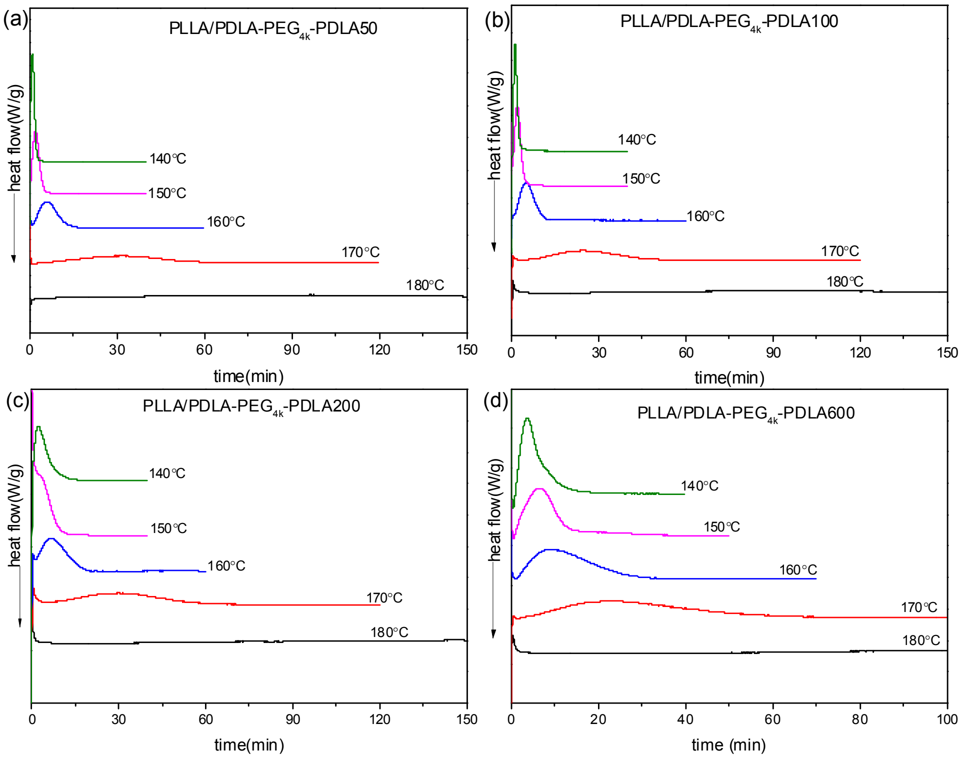

Figure S7. Isothermal crystallization curves of the blends with different PDLA blocks at different temperatures.

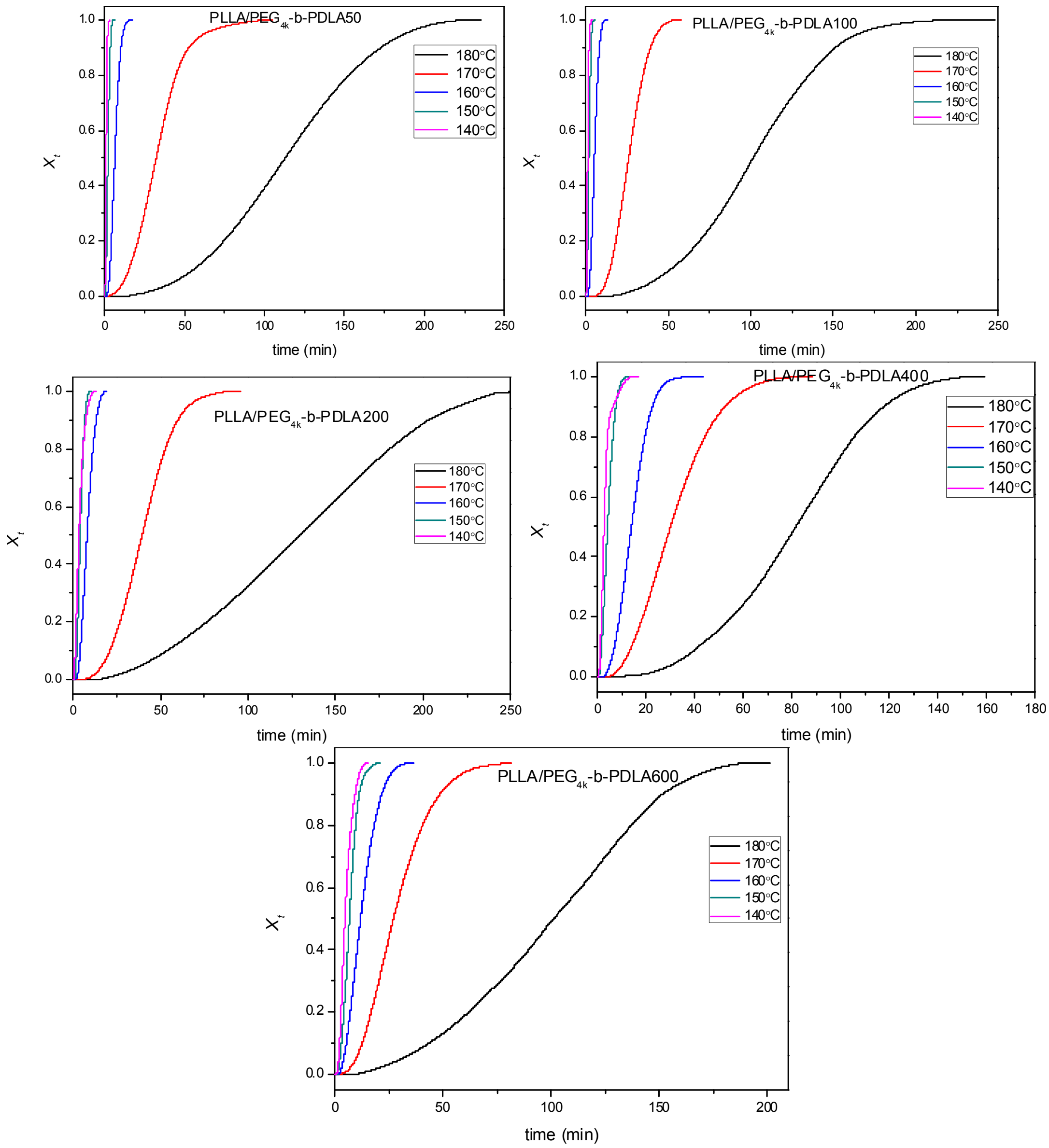

Figure S8. Variation of X_t with crystallization time *t* of the blends with different PDLA blocks at different crystallization temperatures.

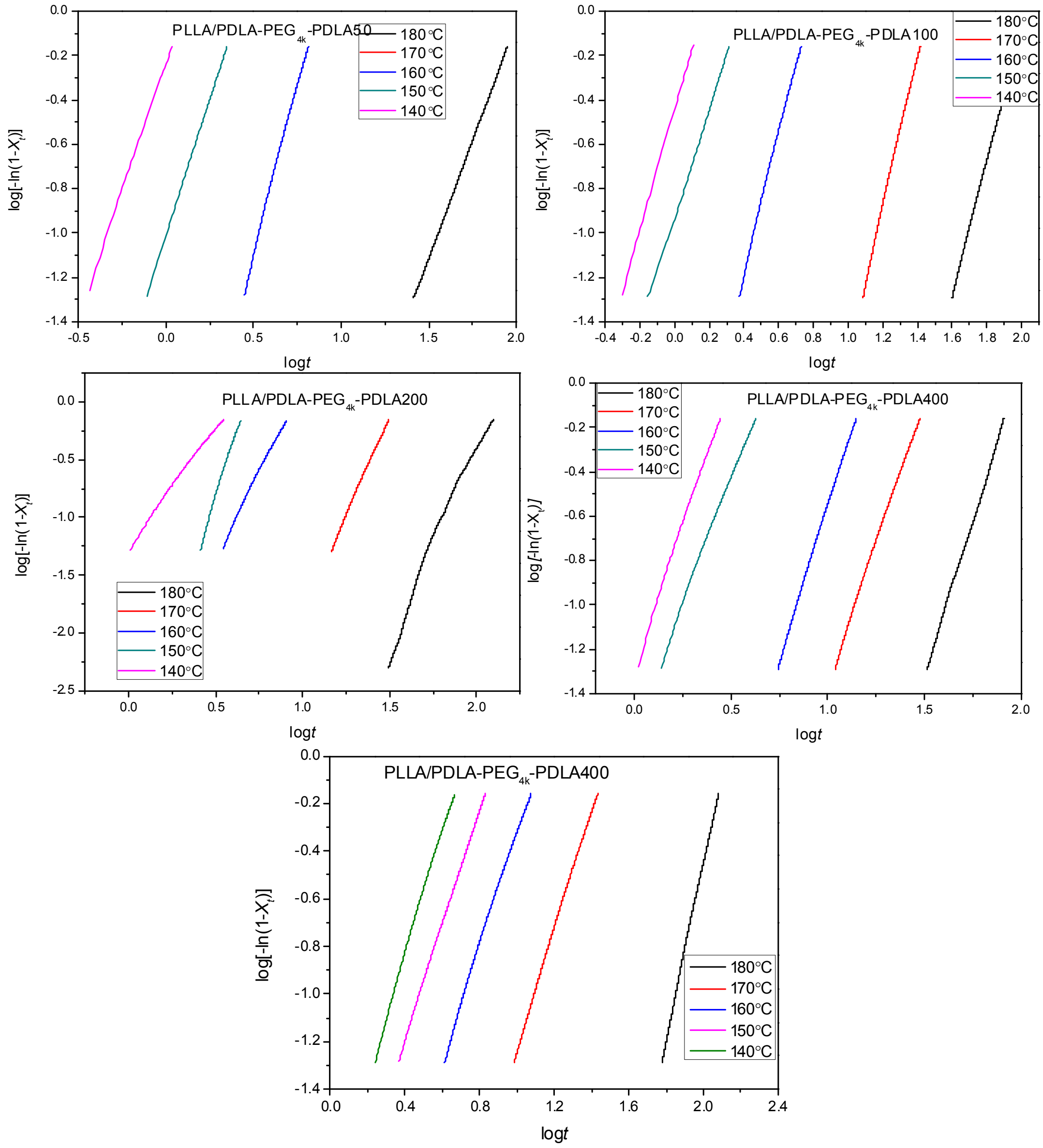

Figure S9. The plots of log[−ln(1−X_t)] versus log*t* at different crystallization temperatures.

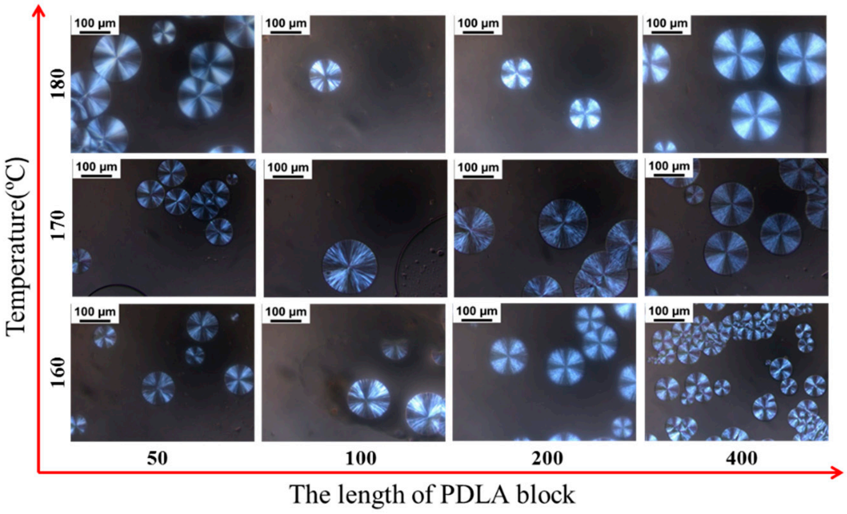

Figure S10. POM images of the blends with different PDLA blocks isothermally crystallized at different temperatures.
